# Linkage between fecal androgen and glucocorticoid metabolites, spermaturia, body weight and onset of puberty in male African lions (*Panthera leo*)

**DOI:** 10.1371/journal.pone.0217986

**Published:** 2019-07-03

**Authors:** Sarah B. Putman, Janine L. Brown, Craig Saffoe, Ashley D. Franklin, Budhan S. Pukazhenthi

**Affiliations:** 1 Center for Species Survival, Smithsonian Conservation Biology Institute, Front Royal, VA, United States of America; 2 George Mason University, Fairfax, VA, United States of America; 3 National Zoological Park, Washington, DC, United States of America; 4 AZA Reproductive Management Center, Saint Louis Zoo, St. Louis, MO, United States of America; Centre for Cellular and Molecular Biology, INDIA

## Abstract

There is limited physiological information on onset of puberty in male lions. The aim of this study was to use longitudinal non-invasive monitoring to: 1) assess changes in steroid metabolite excretory patterns as a function of age and body weight; 2) determine correlations between fecal androgen (FAM) and glucocorticoid (FGM) metabolite concentrations; and 3) confirm spermiogenesis non-invasively through urinalysis. Specifically, FAM and FGM metabolites were analyzed in samples collected twice weekly from 21 male lions at 17 institutions (0.9–16 years of age) for 3.8 months– 2.5 years to assess longitudinal hormone patterns. In addition, body weights were obtained approximately monthly from 10 individuals at five zoos (0.0–3.0 years), and urine was collected from six males at two facilities (1.2–6.3 years) and evaluated for the presence of spermatozoa. An increase in overall mean FAM occurred at 2.0 years of age, at which point concentrations remained similar throughout adulthood. The onset of puberty occurred earlier in captive-born males (<1.2 years of age) compared to wild-born counterparts (<2.5 years of age). Additionally, males in captivity gained an average of 7.3 kg/month compared to 3.9 kg/month for wild males over the first 2–2.5 years of age. Sperm (spermaturia) was observed in males as young as 1.2 years in captivity compared to 2.5 years in the wild (ejaculates). There was no difference in FAM or FGM concentrations with regards to age or season. Overall, this study demonstrates that: 1) captive male lions attain puberty at an earlier age than wild counterparts; 2) onset of puberty is influenced by body weight (growth rate); and 3) spermiogenesis can be confirmed via urinalysis. Knowledge about the linkage between body weight and onset of puberty could facilitate improved reproductive management of *ex situ* populations via mitigating the risk of unintended breedings in young animals.

## Introduction

African lions (*Panthera leo*) have been kept in menageries and for public display since the Roman Era, and in the 21^st^ century an estimated 750 lions were maintained in captivity globally [1]. Lions historically have bred well in captivity compared to other felids [2–6], limiting the incentive for conducting biological research. Most studies are based on behavioral observations of wild lions [7,8]; few included biological data [9–11]. Consequently, little is known about longitudinal gonadal and adrenal hormonal patterns or how they are associated with the onset of puberty in male lions.

Puberty is defined as the process of acquiring reproductive competence [12]. In males, puberty is characterized by the development of secondary sexual characteristics, display of mounting behaviors, appearance of sperm in urine or ejaculate, and/or ability to ejaculate [12]. In addition to genetic and environmental factors, there is increasing evidence that nutritional status during early development influences maturation of the hypothalamic-pituitary-gonadal axis, resulting in earlier onset of puberty and sexual maturity. Extensive literature is available on the onset of puberty in livestock species, including cattle [13,14]. Among wildlife species, onset of puberty has been studied in species such as raccoons (*Procyon lator*) [15]), baboons (genus Papio) [16], wild boars (*Sus scrofa*) [17], American black bears (*Ursus americanus*) [18], polar bears (*Ursus maritimus*) [19], cheetahs (*Acinonyx jubatus*) [20], and European badger (*Meles meles*) [21].

Previous studies have characterized lions into reproductive life stages using behaviors and social dynamics. The average ages across these studies for wild male lions are: cub (0–2.2 years), subadult (2.2–4.5 years), adult (4.5–10 years), and aged (>10 years) [11,22–24]. Yet, these distinctions are more a function of social structure and morphology than reproductive capability. Using behaviors and dimorphic physiological changes, most sources agree that puberty occurs in male cubs at or after 2.2 years of age (~26 months) [10,25], which corresponds to the approximate age that they are expelled from their natal pride [7,22,26]. By contrast, in most zoos, young males are housed together and recommended for breeding when they are over 3 years of age [27]. The age span of males that have successfully bred in captivity ranges from < 2 to 15.1 years [28]. Furthermore, lions in captivity appear to gain body weight faster than their wild counterparts [10], but there is no physiological or hormonal evidence to determine if the animals attain puberty earlier. Therefore, the objectives of this study were to: 1) use longitudinal non-invasive monitoring to assess changes in steroid metabolite excretory patterns as a function of age and body weight; 2) determine correlations between fecal androgen (FAM) and glucocorticoid (FGM) metabolite concentrations; and 3) confirm spermiogenesis non-invasively through urinalysis.

## Materials and methods

### Ethics statement

Animal-related protocols were conducted with the approval of the Smithsonian National Zoological Park’s Animal Care and Use Committee. Routine noninvasive collection of fecal and urine samples did not warrant additional institutional Animal Care and Use Committee approvals nor did it affect the daily routine of animals used in this study.

### Study animals

A total of 26 male African lions located at 18 AZA-accredited institutions, ranging in age from <1 month to 16.0 years of age were included in the study (Table 1). Twenty-one animals were used for reproductive (hormone) assessment, 11 males were used for body weight measurements, and six males were used for urinalysis. Males were divided into four reproductive life stage age groups: peripubertal, 0.91–1.99 years (n = 5); subadult, 2–2.99 years (n = 5); adult, 3–10.99 years (n = 12); and aged, > 11 years (n = 5). In instances where the data for an individual spanned more than one age group, separate values were calculated for each age group. On average, animals were fasted <1 days/week and provided bones 1.5 days/week. Forty-four percent of the population was fed solely horsemeat (Nebraska Brand, Central Nebraska Packing, Inc. North Platte, NE) while the remaining animals were fed either beef or a combination of the two. All individuals had ad-libitum access to water. Furthermore, all animals in the study were allowed daily outside access for at least 1 hour, as long as temperatures remained above freezing.

**Table 1:** Demographics of male lions included in this study.

		Reproductive assessment (N = 21)						Body weight measurements (N = 11)				Urinalysis (N = 6)		
Facility	SB	Age group*	Age at start of sample collection (yr)	Age at end of sample collection (yr)	Collection duration (mo)	Other pridemates (Y/N)	Proven breeder (Y/N)	Age at start of sample collection (yr)	Age at end of sample collection (yr)	Collection duration (mo)	Other pridemates (Y/N)	Age at start of sample collection (yr)	Proven breeder (Y/N)	Other pridemates (Y/N)
**Birmingham Zoo**	420	P						0.1	1.4	14.9	2.3	1.2	N	2.3
** **	422	P						0.1	1.4	14.9	2.3	1.2	N	2.3
**Blank Park Zoo**	Generic #1	Ag	12.5	13.4	10.8	0.2	N							
**Brookfield Zoological Park**	263	S, Ad	2.9	3.5	7.2	0.1	N							
**Cheyenne Mountain Zoological Park**	173	Ag	13	13.8	9.6	1.5	Y							
**Denver Zoological Gardens**	236	P						0	1.1	12.7	3.3			
** **	234	Ad	6.8	7.9	13.2	2.3	Y							
** **	136	Ad	6.4	7.4	12.5	2.3	N							
** **	137	Ad	6.4	7.4	12.5	2.3	N							
** **	243	P						0	1.3	15.5	4.6			
**Henry Villas Zoo**	221	Ag	11.2	12.6	16.8	0.1	Y							
**Indianapolis Zoo**	75	Ag	14.8	16	14.4	0.2	Y							
**Knoxville Zoological Garden**	146	Ad	5.9	6.9	12	0.2	N							
**Lee Richardson Zoo**	243§	S, Ad	2.6	3.6	12	2.3	N							
**Milwaukee County Zoological Garden**	225	Ad	3.5	4.7	14.4	0.1	N							
**North Carolina Zoological Park**	148	Ad	9.6	10.7	13.2	0.1	Y							
**Oklahoma City Zoo**	64	Ag	11.3	11.6	3.8	0.2	N							
**Potawatami Zoo**	Generic #2	Ad	7.1	8.1	12	1	N							
**Riverbanks Zoological Park**	215	P, S, Ad	1.6	4	28.8	0.2	Y							
** **	313	P						0	2.5	30.2	2.5			
** **	334	P						0	2.5	30.2	2.5			
**Santa Barbara Zoological Gardens**	128	Ad	6.3	6.7	4.8	0.1	Y							
**Smithsonian National Zoological Park**	248	S, Ad	2	4.3	28.6	0.2	N	1.2	2.1	10	0.2	6.3	Y	3.6
** **	409	P	1	2	12.2	3.6	N	0.1	2.5	29.4	3.6	1.5	N	3.6
** **	411	P	0.9	1.8	10.7	3.6	N	0	1.7	20.4	3.6	1.4	N	3.6
** **	412	P	0.9	1.8	10.7	3.6	N	0	1.7	20.4	3.6	1.4	N	3.6
**Virginia Zoological Park**	386	P	1.5	1.9	4.8	1.2	N	0.1	1.8	20.4	1.2			
**Wildlife Safari Inc**	386+	S	1.9	2.6	8.4	0.1	N							

*Age Groups: P = Peripubertal, S = Subadult, Ad = Adult, Ag = Aged; Some animals contributed to more than one age group in this study. Animals include SB263 (subadult and adult), SB243 (subadult and adult), SB215 (peripubertal, subadult, and adult), and SB248 (subadult and adult).

^§^SB243 transferred from Denver Zoo to Lee Richardson Zoo during the study and hence was counted as the same animal.

^+^SB386 tranfserred from Virginia Zoological Park to Wildlife Safari, Inc during the study and hence was counted as the same animal.

### Sample collection and processing

#### Sample collection

On average, fecal samples were collected 2x/week for at least 3.8 months and as long as 2.5 years (Table 1, N = 21) and frozen within 24 hours in plastic zip-top bags. Urine samples (>1 ml; N = 6) for spermatozoal assessment were aspirated from concrete flooring opportunistically from six male lions at two facilities: a 6.3-year proven male that served as a positive control; three ranging in age from 1.4–1.5 years; and pooled samples from a pair of siblings, 1.2 years old. Urine samples were placed in polypropylene tubes and frozen within 12 hours of being voided.

#### Fecal processing and steroid hormone extraction

Fecal samples were processed and hormone metabolites extracted as previously described [29]. Briefly, samples were dried in a lyophilizer (VirTis Ultra 35XL, SP Scientific, Warminster, PA), powdered, sifted, and 0.20 ± 0.02 g was weighed into 16x125 mm glass tubes (Fisherbrand, Thermo Fisher, Pittsburgh, PA). Five ml of 90% ethanol:10% de-ionized water was added to each sample along with ~20,000 dpm ^3^H–cortisol tracer (NEN Radiochemicals, Perkin Elmer, Boston, MA) to determine procedural loss. For 20 minutes, samples were boiled in a 95°C waterbath and maintained at a 5-ml volume with the addition of 100% ethanol as needed. Samples were then centrifuged at 500 x *g* for 20 minutes (Sorval RC 3C Plus, Kendro Laboratory Products, Newtown, CT), the supernatant recovered and 5 ml of 90% ethanol:10% de-ionized water added to the pellet, which was vortexed (pulse rate 1/second, speed 65; Glas-Col, Terre Haute, IN) for 30 seconds. Samples were centrifuged (15 minutes, 500 x *g*), and the supernatants combined and dried down under forced air. One ml of 100% methanol was then added to dried sample extracts, evaporated to dryness, and reconstituted in 1 ml of preservative-free buffer (0.2 M NaH_2_PO_4_, #S8282; 0.2 M Na_2_HPO_4_, #S7907, Sigma Aldrich, St. Louis, MO; 0.15 M NaCl, #S271, Fisherbrand; pH 7.0). After vortexing for 15 seconds, samples were placed in an ultrasonic cleaner water bath (Cole Parmer Instrument Company, Vernon Hills, IL) for 15 minutes. Sample extracts were further diluted in buffer as needed: 1:10–1:50 for glucocorticoids and 1:50–1:250 for androgens. All sample extracts and dilutions were stored in polypropylene tubes at -20°C until analysis. Average recovery of the ^3^H-cortisol tracer was 82 ± 0.26% (mean ± standard error of the mean; SE).

#### High pressure liquid chromatography (HPLC)

High pressure liquid chromatography (Varian ProStar; Varian Analytical Instruments, Lexington, MA) of pooled fecal extracts was performed to characterize steroid hormone metabolites similar to that described previously [30]. Briefly, six fecal samples were extracted and the supernatants pooled, dried down, reconstituted in 1 ml methanol, passed through a syringe filter (13 mm, 0.2 μm pore size, #6789–1302, Whatman, Inc. Clifton, NJ) and dried under forced air. The pooled extract was reconstituted in 0.5 ml PBS (0.03 M Na_2_HPO4, 0.02 M NaH_2_PO4, 0.15 M NaCl, 0.002 M NaN_3_, #S2002, Sigma Aldrich; pH: 5.0), filtered through a C18 Spice cartridge (Analtech, Inc., Newark, DE), and evaporated to dryness. Approximately 14,000 dpm of radioactive tracers (^3^H-testosterone, ^3^H-cortisol and ^3^H-corticosterone) were added to the appropriate pooled sample as chromatographic markers and dried again. The extract was reconstituted in 0.3 ml methanol, sonicated for 5 minute, and 0.05 ml was loaded onto a reverse-phase C18 HPLC column (Agilent Technologies, Santa Clara, CA). For testosterone, the sample was separated using a 45% isocratic acetonitrile:water solution over 80 minute (1 ml/minute flow rate, 0.33 ml fractions). For cortisol, the sample was separated using a 20–80% linear gradient of methanol:water over 80 minute (1 ml/minute flow rate, 1 ml fractions). A multi-purpose β-radiation scintillation counter (LS 6500, Beckman Coulter, Brea, CA) was used to evaluate a 0.05-ml aliquot of each fraction; the remaining volume of each fraction was dried and resuspended in 0.25 ml preservative-free phosphate buffer. Each fraction was then analyzed in singlet using the appropriate EIA and the retention times of chromatographic standards and immunologic activity were compared.

#### Steroid hormone analysis

Single-antibody enzyme immunoassays (EIAs) [31], utilizing polyclonal antibodies to testosterone (R156/157) and cortisol (R4866; C.J. Munro, University of California, Davis, CA) and the corresponding horseradish peroxidase ligands (lot: 2005/2006, SCBI, Front Royal, VA) [32] were used to measure fecal androgen (FAM) and fecal glucocorticoid (FGM) metabolites, respectively. The cross-reactivities for R156/157 were: testosterone 100.0%, 5α-dihydrotestosterone 57.4% and all other compounds cross-react with the antibody < 1.0% (C.J. Munro, personal communication). The cross-reactivities for R4866 were: cortisol 100.0%, prednisolone 9.9%, prednisone 6.3%, cortisone 5.0%; all other compounds cross-react with the antibody < 1.0% [33]. The standard curve ranges were: testosterone: 0.47–12.00 ng/ml; cortisol: 0.78–20.00 ng/ml. Briefly, antibodies in a coating buffer (0.015 M Na_2_CO_3_, #S2127; 0.035 M NaHCO_3_, #S8875, Sigma Aldrich, St. Louis, MO; pH 9.6) were adsorbed to flat-bottomed, high-binding 96-well microtiter plates (Nunc-Immuno, Thermo Fisher) and incubated ≥ 8 hours at 4°C. The plates were washed five times (0.05% Tween 20, #P1379, Sigma Aldrich; in 0.15 M NaCl solution) followed by the addition of 0.05 ml standard (testosterone: #46923; hydrocortisone: #H4001, Sigma Aldrich), internal control or diluted sample in duplicate, and then 0.05 ml horseradish peroxidase solution. Plates were washed five times after an incubation of 2 hours for testosterone and 1 hour for cortisol at RT, and 0.1 ml ABTS solution (0.04 M ABTS diammonium salt, #0400, Amresco, Solon, OH; 0.5 M H2O2 #BP2633, Fisherbrand, in 0.05 M citric acid buffer, #C0759, Sigma Aldrich; pH 4.0) was added to each well. All EIAs were read on a microplate reader (MRX, Dynex Technologies, Chantilly, VA) at 405 nm (ref. 490 nm) when the optical density (OD) of the 0.00 ng/ml standard was ~1.00 (range 0.90–1.10). Data are reported as μg/g dry feces. The intra-assay coefficients of variation (CV) between duplicates for all samples were <10%. Inter-assay CV for two (high and low concentration) internal controls were monitored on each assay. The CVs of the controls were: testosterone: 8.9% and 10.8% (n = 100); and cortisol: 5.5% and 10.2% (n = 113).

Enzyme immunoassays were validated by demonstrating: 1) parallelism between standard curves and serially diluted fecal extracts; 2) recovery of hormone standard added to fecal extracts; and 3) correlation of hormone data with physiological events. Two-fold serial dilutions of samples were parallel to the standard curve for each EIA. For testosterone, the slopes of the standards and the sample dilutions were -12.18 and -13.82, respectively (r = 0.99), and for cortisol were -11.47 and -11.66, respectively (r = 0.99). For testosterone, the slope of hormone recovery was y = 0.82x + 4.69 (r = 0.99) when exogenous steroid was added to pooled fecal extract diluted 1:50. The slope of cortisol added to pooled fecal extracts (diluted 1:10) was y = 1.13x + 2.85 (r = 0.99). Increases in FAM in two male lions (SB409 and SB248) associated with mane growth, a secondary sexual characteristic controlled by testosterone [34] showed the biological validity of the testosterone EIA (S1 Fig). To validate the FGM EIA, FGM was measured in a male that exhibited lethargy and hematuria. Fecal FGM in samples collected over 2 weeks prior to the onset of symptoms (0.29 ± 0.04 μg/g; N = 6) and the 2 weeks after symptoms resolved (0.28 ± 0.02 μg/g; N = 6) were lower than the average FGM concentration during the duration of the illness (0.41 ± 0.02; N = 8; *F*_2,17_ = 8.75 *P* = 0.002; Tukey, before: *P* = 0.01 and after: *P* = 0.005 treatment).

### Urine processing and evaluation

Sample processing and evaluation was modified from human protocols [35,36]. Briefly, urine samples were thawed at RT and 1-ml aliquots were spun at 1,100 x *g* for 7 minutes (MiniSpin Plus, Eppendorf North America, Hauppauge, NY). After centrifugation, the supernatant was aspirated and < 0.01 ml of the pellet was examined under 400x magnification (Olympus BH2 microscope, Olympus Corporation, Center Valley, PA) for the presence of spermatozoa. Each pellet was analyzed a maximum of three times for the presence of sperm, after which if no spermatozoa were observed, the sample was categorized as sperm negative.

#### Body weight measurement

Body weights of captive lions from <1–30 months were obtained at least monthly for 11 individuals at six institutions for at least 12 months. Animals <4 months of age were weighed inside a pre-tared plastic tube on a platform scale; after 4 months, they were weighed either on a platform scale during training sessions or inside a squeeze cage. Weights from wild-born cubs (N = 26) ranging in age from <1–30 month were derived from data collected in Kruger National Park, South Africa [10,37].

### Data analysis

An iterative process was utilized to determine baseline concentrations for steroid hormones in all study animals individually [38]. Briefly, data were deleted if the concentration was greater than 2 times the standard deviation (SD) above the mean. This process was replicated until no further data points could be removed. The resulting mean was considered the baseline concentration for that hormone and data points greater than 2 SD above the mean were regarded as peak concentrations.

For individual study animals, FAM and FGM hormone data were calculated for overall, baseline and peak means (μg/g dry feces ± SE). Repeated measures analysis of covariance (ANCOVA) utilizing a compound symmetric covariance matrix structure and post-hoc Tukey HSD tests established the differences in FAM and FGM concentrations among age groups and across seasons. Correlations between FAM and FGM were calculated using Pearson’s correlation coefficient; analyses were performed on all data, by age group, and within individual.

For seasonality, individual mean and baseline hormone concentrations were calculated for each season and then seasons were averaged by age group to obtain a final mean. For males with more than 1 year of sample collection, only one complete year was utilized. The dates for each of the seasons are as follows: Spring, March 21 –June 20; Summer, June 21 –September 20; Fall, September 21 –December 20; and Winter, December 21 –March 20.

Body weights of wild lion cubs in Kruger National Park were obtained from previously published data [10,37] and averaged by month, as were body weight measurements from zoo-born cubs. Monthly differences in body weight between locations were calculated using ANCOVA with post-hoc Tukey HSD tests. Linear regression slopes were calculated for the overall weights of wild and captive cubs.

A *P* < 0.05 α-level was used to determine statistical significance. In analyses where individual animals were repeatedly sampled, data were either blocked by individual or analyzed as repeated measures. The Kenward-Roger adjustment for degrees of freedom was used when employing repeated measures analyses. Analyses were conducted using SAS v. 9.3 (SAS Institute Inc., Cary, NC, USA).

## Results

### HPLC

All of the immunoactivity in fractions evaluated using the testosterone EIA was present in fractions 9–15, and no activity was associated with the radioactive testosterone tracer (fraction 32). Analysis of fractions using the cortisol EIA indicated 80% of immunoactivity was at fraction 40, with a smaller peak (20%) observed at fraction 45, corresponding to the retention times of ^3^H-labeled cortisol (fractions 40–41) and corticosterone (fractions 45–46), respectively.

### Reproductive and adrenal hormone patterns

**Fig 1:**
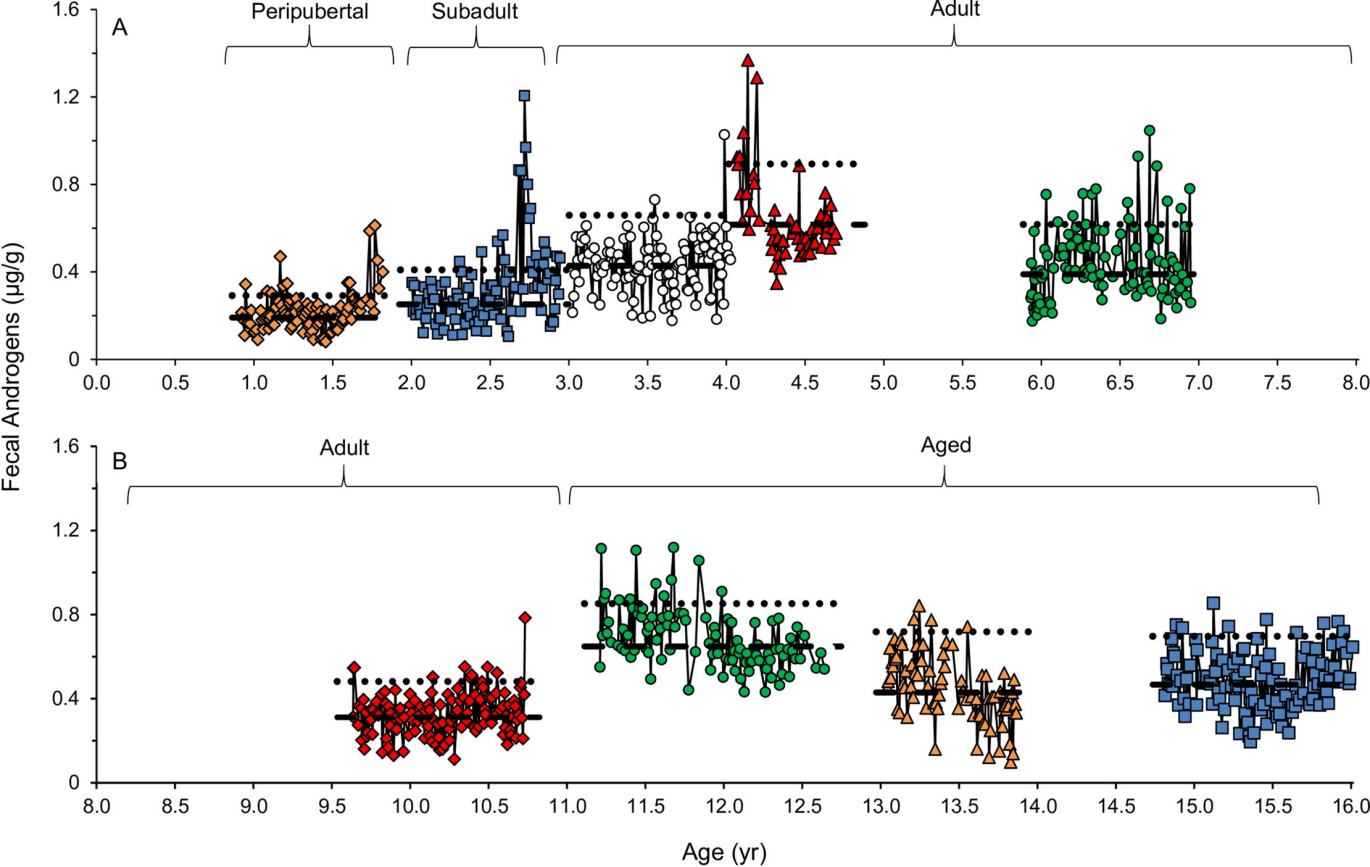
Representative fecal androgen metabolite (μg/g) profiles of nine males of diverse ages. Panel A; Peripubertal (0.9–2 yr): SB411 (orange diamonds), Subadult (2–2.99): SB248 (blue squares) and Adult (3–10.99): SB215 (open circles), SB225 (red triangles) and SB146 (green circles). Panel B; Adult: SB148 (red diamonds) and Aged (>11 yr): SB221 (green circles), SB173 (orange triangles) and SB75 (blue squares). Baseline (dashed line) and threshold (dotted line) concentrations were calculated for each male.

There was a difference in overall mean (*F*_3,16.6_ = 8.25 *P* < 0.01), baseline (*F*_3,10.8_ = 9.71 *P* < 0.01) and peak (*F*_3,10.7_ = 5.83 *P* = 0.01) FAM concentrations among the age groups (Table 2). The overall mean FAM concentrations were similar among subadult, adult and aged groups, (subadult and adult: *P* = 0.99; subadult and aged: *P* = 0.36; adult and aged: *P* = 0.13), while the peripubertal group was consistently lower (peripubertal and subadult: *P* = 0.02; peripubertal and adult; *P* = 0.01; peripubertal and aged: *P* < 0.001). Peripubertal baseline FAM was lower than all other age groups (peripubertal and subadult: *P* = 0.03; peripubertal and adult: *P* = 0.01; peripubertal and aged: *P* = 0.001), the subadults were lower than the aged group (*P* = 0.03), yet the subadult and adult and the adult and aged age groups were similar (*P* = 0.79 and *P* = 0.05, respectively). The peak FAM concentrations were similar among all age groups apart from the peripubertal group (peripubertal and subadult: *P* = 0.01; peripubertal and adult: *P* = 0.02; peripubertal and aged: *P* = 0.04; subadult and adult: *P* = 0.98; subadult and aged: *P* = 0.96; adult and aged: *P* = 0.86). Fig 1 illustrates representative profiles of FAM patterns in peripubertal (0.9–2 years; Panel A), subadult (2–2.99 years; Panel A), adult (3–10.99 years; Panels A and B) and aged individuals (> 11 years; Panel B). Concentrations of FGM were similar among age groups (Table 2; overall: F_3,9.04_ = 2.76 *P* = 0.10; baseline: F_3,9.11_ = 3.02 *P* = 0.09; peak: F_3,19.5_ = 0.44 *P* = 0.73). There was a weak to moderate positive correlation between FAM and FGM concentrations across all age groups (r = 0.39, P < 0.001), within each age group (range from r = 0.24 to 0.40, P < 0.001; Table 3), and within 15 of 21 individual male lions (S1 Table).

**Table 2:** Overall, baseline and peak mean (± SE) concentrations of fecal androgen and glucocorticoid metabolites by age group.

Age Group	Fecal Androgens (μg/g)			Fecal Glucocorticoids (μg/g)		
	Overall	Baseline	Peak	Overall	Baseline	Peak
Peripubertal	0.28 ± 0.04^a^	0.25 ± 0.04^a^	0.66 ± 0.05^a^	0.12 ± 0.01^a^	0.11 ± 0.01^a^	0.36 ± 0.08^a^
Subadult	0.49 ± 0.07^b^	0.41 ± 0.04^b^	1.02 ± 0.15^b^	0.17 ± 0.01^a^	0.14 ± 0.02^a^	0.60 ± 0.16^a^
Adult	0.46 ± 0.02^b^	0.42 ± 0.02^b^^,^^c^	1.02 ± 0.07^b^	0.23 ± 0.03^a^	0.19 ± 0.02^a^	0.75 ± 0.20^a^
Aged	0.60 ± 0.06^b^	0.56 ± 0.05^c^	1.16 ± 0.16^b^	0.24 ± 0.04^a^	0.22 ± 0.04^a^	0.55 ± 0.12^a^

^a,b,c^ Within column, values with different superscripts are significantly different (*P* < 0.05).

**Table 3:** Correlations between fecal androgen and glucocorticoid metabolites.

Age Group	Pearson’s Correlation Coefficient		
	N	r	*P*
Overall	2180	0.3852	<0.001
Peripubertal	416	0.2380	<0.001
Subadult	286	0.3142	<0.001
Adult	1110	0.3296	<0.0001
Aged	368	0.3959	<0.001

There was no influence of season on overall, baseline or peak concentration means of FAM (mean: F_3,57.5_ = 1.21 *P* = 0.31; baseline: F_3,57.3_ = 1.55 *P* = 0.21; peak: F_3,57.8_ = 1.53 *P* = 0.22) or FGM (overall: F_3,55.2_ = 1.92 *P* = 0.14; baseline: F_3,57.1_ = 2.32 *P* = 0.08; peak: F_3,57.5_ = 1.05 *P* = 0.38).

### Urinalysis

Spermatozoa were present in all but one urine sample (S2 Fig) collected (83%) from male lions ranging in age from 1.2–6.3 years, including a proven male (SB248) that served as a positive control (Table 4).

**Table 4:** Detection of spermatozoa in urine from lions of varying ages.

SB	Age at collection (years)	Sperm present	Weight (kg)
248	6.3	Yes	177
409	1.5	Yes	143
411	1.4	Yes	145
412	1.4	No	145
	1.5	Yes	156
420/422	1.2	Yes	105

### Body weight

**Fig 2:**
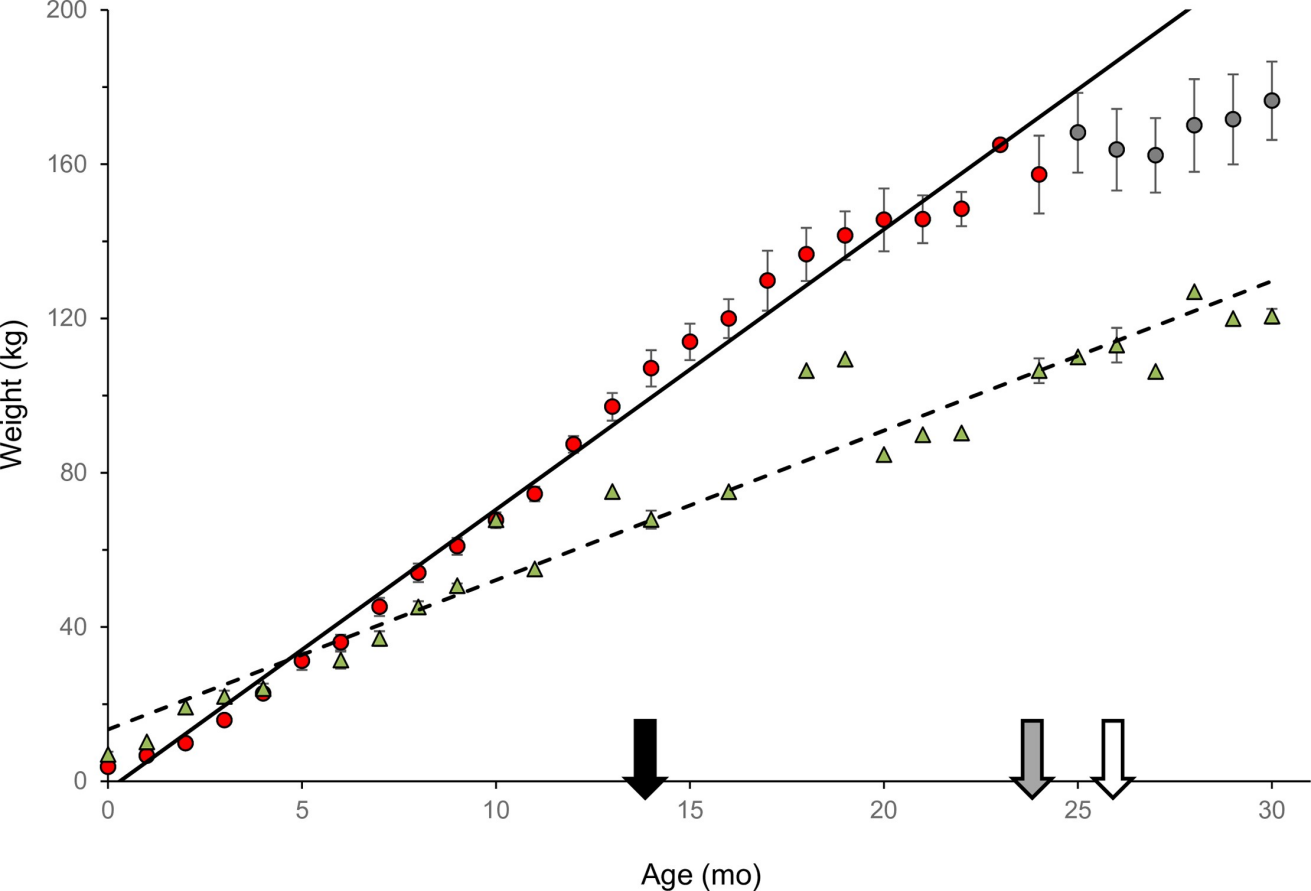
Monthly average (± SE) body weights (kg) of captive (circles, N = 11) and wild (green triangles; adapted from Smuts et al., 1980; N = 26) male lions through 30 mo (2.5 yr) [10]. Red circles denote months when captive males were actively gaining weight and grey circles represent months where the rate of weight gain slowed. Trend lines are solid for captive lions and dashed for wild lions. The black arrow indicates the earliest age, 14 mo (1.2 yr), and body weight, 105 kg, at which spermatozoa were present in captive male urine (n = 2). The grey arrow denotes when spermatogenesis should be occurring in wild lions based on the age, 24 mo (2.0 yr), at which they reach ~105 kg and the open arrow is the age (26 mo, 2.2 yr) and body weight (113 kg) when males reach puberty in the wild based on behavioral observations [10] and the earliest age spermatozoa were found in seminiferous tubules in wild males [16]. (Modified data on wild lions by Smut et al., used with permission from John Wiley and Sons) [10].

Average monthly body weights differed between captive and wild-born cubs (Fig 2, F_1,188_ = 149.35 *P* < 0.001). Wild-born cubs weighed more than captive-born counterparts at 0–2 months (Month 0: *P* < 0.001; Month 1: *P* < 0.001; Month 2: *P* = 0.006), had similar body weights at 3–5 months (Month 3: *P* = 0.10; Month 4: *P* = 0.73; Month 5: *P* = 0.24), but weighted less between 6–30 months (Month 6: *P* = 0.006; Months 7–24: *P* < 0.001). Using regression analysis, captive males gained 7.27 kg/month (242.3 g average daily gain, ADG) for the first 24 months, then slowed to 2.66 kg/month (88.7 g ADG) for the next 25–30 months. In comparison, wild males gained an average of 3.88 kg/month (129.3 g ADG) over a 30-month study period.

## Discussion

This study represents the first analysis of longitudinal FAM and FGM patterns in male African lions utilizing a non-invasive approach, identifying the influence of age on testicular and adrenal steroidogenic activity. Urinalysis for spermatozoa presence also proved to be a novel, non-invasive method for determining puberty onset in young lions. Overall results indicate that captive lions are maturing faster than wild counterparts, which was related to differences in growth rates during the first few years of life. These data provide fundamental information regarding male lion gonadal and adrenal function, and how it relates to age and reproductive capacity that can be used by the Lion Species Survival Plan (SSP) and animal care staff to improve captive animal management.

Relevant steroid hormone metabolites were reliably detected in lion fecal samples via EIA. Based on HPLC analysis, none of the immunoactivity was associated with native testosterone, but rather presumably conjugated polar metabolites, which agrees with studies in domestic cats (*Felis catus*), Pallas’ cats (*Otocolobus manul*), Eurasian lynx (*Lynx lynx*), and Iberian lynx (*Lynx pardinus*) [29]. By comparison, the cortisol EIA detected native cortisol as a major source of immunoactivity in HPLC fractions. These results differ from other felids, including clouded leopards (*Neofelis nebulosa*) and cheetahs (*Acinonyx jubatus*) [39], where native cortisol is not a detectable excretory product.

The five age categories in this study (cub, peripubertal, sub-adult, adult and aged) were based on prior studies of wild lions [10,23], but were refined because of our results. In wild males, individuals are considered cubs until they are at least 2 years, but in captivity, individuals <2 years are capable of producing offspring [28]. As a result, cubs were reclassified as <0.9 years. The age range of 0.9–1.99 years was reclassified as peripubertal because of higher body weight s compared to wild lions but lower FAM than captive adults, and the onset of spermaturia indicative of puberty [10,12,23]. Captive males 2–2.99 years of age were classified as subadult to indicate that while they are not yet full adult weight, they are still heavier than their wild counterparts [10,23] and had similar FAM to adult males. The adult age range shifted to include 3-year-old males because they had reached adult weight by that age, and in the wild would be physically capable of gaining and holding control over a pride [26,40], and their FAM was similar to males at the age typical of retaining a pride. Wild males >8–9 years were still classified as adults [23]. Captive males were considered aged starting at 11 years to identify a population rarely observed in the wild and from which no wild hormone data are available [11].

Concentrations of FAM were lower in peripubertal males compared to subadult, adult and aged males in agreement with other felid studies, although the effect of age on gonadal activity has not been studied extensively in these species. In Iberian lynx, young males (2 years old) produced the lowest, while older males (>4 years) excreted the highest FAM concentrations, and results from electroejaculation trials found that even 2-year-old males were spermic, albeit with a higher percentage of abnormal spermatozoa [41]. Likewise, FAM concentrations were lower in juvenile (2 years) compared with adult (3–18 years) Canada lynx (*Lynx canadensis*) [42].

Concentrations of FAM were consistent throughout the year, indicating that captive male lions are not seasonal. Seasonality in testosterone production is more common in felids found in temperate latitudes, such as the Pallas’ cat [43], Canada lynx [42] and Iberian lynx [41] compared to cats from tropical and subtropical climates, like the margay (*Leopardus wiedii*) and tigrina (*L*. *tigrinus*) that do not show seasonal changes in FAM [44]. However, not all cats found closer to the equator are aseasonal; the ocelot (*L*. *pardalis*) [44] and clouded leopard [45] do exhibit changes in testosterone production associated with season.

Concentrations of FGM were similar across age groups and did not vary with season. Furthermore, there was a relationship between FAM and FGM in most males individually, and all age groups showed weak to moderate positive correlations between the two hormones. There are limited data on the relationship between testosterone and cortisol production in felids; most relate to how testosterone changes as a result of anesthesia, administration of ACTH, or other procedures (e.g. clouded leopards and jaguars (*Panthera onca*) [45,46]. Experimental manipulation of cortisol by ACTH and dexamethasone suggest that corticoids can increase testosterone production in arctic ground squirrels (*Spermophilus parryii plesius*) [47]. It is unlikely that the observed correlation in lions is due to the cortisol EIA is crossreacting with FAM because an increase in FGM concentrations was not observed in the young males as they aged and FAM significantly increased.

Longitudinal analysis of androgen production in relation to puberty onset is not well documented in felids. In domestic cats, fecal testosterone was monitored from birth to the weeks leading up to the average age of puberty, and a rise in testosterone was observed in neonates [48]. However, testosterone remained low in subsequent weeks until the individuals were castrated [48]. Recently, Maly et al [20] reported that *ex situ* managed cheetahs reached adult FAM concentrations by 18–24 months of age and body weight by 21 months. They also concluded that based on increases in androgens and body weights, male cheetahs reached puberty at 18–24 months of age. Interestingly, based on the Lion SSP studbook records, males under 2 years of age have sired litters of cubs [28], and the urinalysis results in this study showed that animals with lower testosterone concentrations than average adult males are capable of supporting complete spermatogenesis. It is likely that testosterone concentrations are even lower in males <1 years of age, so that average mean and baseline observed between 1–2 years represents an increase in testosterone capable of initiating spermatogenesis.

A major finding in this study was that captive male lions appear to reach puberty at a younger age than wild counterparts. Based on when males first show mounting behaviors, wild males reach sexual maturity at 2.2 years (26 months), but do not typically breed until they take over a pride at the average age of 4 [26,49] or 5 [23,50] years. Still, individuals as young as 3.3 years have been observed to control prides in the Serengeti and Ngorongoro Crater [11]. In wild lions, males 1.6–1.8 years of age were considered pre-pubertal because they produced lower serum testosterone than young adults and adults, weighed only ~88 kg, and were aspermic [11]. Histologic evaluation of testicular tissue from wild males further demonstrated that the onset of spermatogenesis begins at about 2.5 years of age (range: 2.2–2.8 years) [23]. Our study found that although the peripubertal age group had lower FAM concentrations compared to older age categories, the youngest males that tested positive for spermaturia were 105 kg at 1.2 years of age.

The growth kinetics presented in the present study indicate that captive-born lion cubs develop at a faster rate than wild-born cubs, which could account for the early puberty. Wild cubs are heavier for the first few months after birth, but between 3–5 months of age the growth patterns were the same to our captive cubs. Both captive and wild cubs begin to taste/consume meat a few months after birth and are usually weaned by 0.5 years [22,27,50]. However, after weaning, the plane of nutrition appears to diverge between wild- and captive-born cubs and the ADG rate is no longer synchronous, perhaps a result of feeding captive cubs meat daily. There is substantial variability in growth rate in wild African lions [22], and the rate at which lion cubs grow is correlated with food availability once they are weaned [50,51]. Cubs are dependent on adults for food [7,22], but when prey is scarce, young lions go without food for extended periods [50–52] and starvation is a common cause of death at that age [7,22,53]. Captive cubs likely experience an early onset of puberty and reach adult body weight earlier as a result of the consistent feedings they are provided [22,50,54]. For most mammals, the onset of puberty is associated with attaining a threshold body weight [55–59] and acquiring adequate fat reserves [60]. Under-nutrition can delay onset of puberty while over-conditioned animals often attain puberty at an earlier age.

The minimum body weight for triggering onset of puberty has not been established for African lions. The lightest wild males in Kruger National Park with spermatozoa in their seminiferous tubules weighed 110 kg (n = 2) [10]. Captive males reached ≥115 kg at ~1.3 years, compared with 2.2 years of age in the wild when they are reported to attain puberty. Males SB420 and SB422 were 105 kg at 1.2 years of age when spermatozoa were detected in their urine, corroborating Smuts’ findings based on lion weight. In the wild, growth slows between 2–3 years, but weight gain continues until lions reach a maximum weight around 6 years of age [22,50]. By contrast, captive males reached an adult weight by 3 years [27]. Additionally, the onset of mane development has been reported to occur earlier in captive males, another indicator that androgen production increases at a younger age than in wild males [61]. The observation of spermaturia in captive lions at 14 months of age (1.2 years) indicates that spermatogenesis and spermiogenesis were ongoing before that. Many zoos have programs that include training various species to urinate on command. Being able to identify when a young male has reached puberty through urine analyses would allow zoos to better manage them (separating males and females) and avoid inbreeding.

In summary, this study provides the first extensive study of androgen and glucocorticoid production in male lions. With the addition of urinalysis for spermaturia onset and body weight measurements, we are now better able to identify the onset of puberty in young males. These approaches could serve as a model for understanding onset of puberty in other felid species. It also may be informative to examine the reproductive hormone profile as well as urine samples before 1 year of age to monitor the increase in FAM prior to the onset of spermatogenesis and spermiogenesis. While spermaturia implies the onset of spermatogenesis and spermiogenesis, electroejaculations would provide more information on ejaculate and spermatozoal quality. Over 20 years ago, Brown et al. [11] remarked on the dearth of knowledge on pubertal changes in semen quality in felids; until now, little new information has been added. Future studies could show how felid ejaculate traits are impacted by age or body weight approaching puberty, which might improve management of young felids to avoid inbreeding. Because of declining wild lion populations [62,63], the possibility exists that captive insurance populations may be necessary to conserve the species [64,65]. Overall, results of this study reconfirm a lack of male reproductive seasonality, demonstrates a link between body weight and onset of puberty, as well as validates the utility of urinalysis for assessing reproductive status in male lions. Furthermore, findings add to the information that could be useful for animal managers to improve reproductive management of lions in captivity.

## Supporting information

S1 Table Correlations between fecal androgens and glucocorticoids for all individuals in the study. (DOCX)

S1 Fig Secondary sexual characteristic mane development in two male African lions in relation to their fecal androgen hormone profiles. SB409, orange circles; SB248, green triangles. Black arrows point to the age/hormone concentration when the photo was taken. Red arrow indicates when spermatozoa was found in SB409’s urine. SB409 photos courtesy of Karen Shilling, SB248 photos courtesy of Budhan Pukazhenthi and Smithsonian National Zoological Park.
(TIF)

S2 Fig Presence of spermatozoa (arrows) in urine from pre-pubertal (<1.5 years) male lions. 100x magnification.
(TIF)

S1 File Data for male data averaged for year, age, and season. (XLSX)

S2 File Data averaged for year, age, and season. (XLSX)
