# Supplementary material for: Linkage between fecal androgen and glucocorticoid metabolites, spermaturia, body weight and onset of puberty in male African lions (Panthera leo)
Source: PLoS One. 2019 Jul 3;14(7):e0217986. doi: 10.1371/journal.pone.0217986 (PMC6609010; doi:10.1371/journal.pone.0217986)
Supplement: S1 Table — (DOCX) [file pone.0217986.s001.docx]

**S1 Table.** Correlations between fecal androgens and glucocorticoids for all individuals in the study.

| **Age Group** |  | **Pearson’s Correlation Coefficient** | | |
| --- | --- | --- | --- | --- |
|  | **SB** | **N** | **r** | ***P*** |
| All |  | 2180 | 0.3852 | <0.001* |
| Peripubertal | Overall | 416 | 0.2380 | <0.001* |
|  | 215 | 41 | 0.0128 | 0.9365 |
|  | 386 | 53 | 0.1122 | 0.4239 |
|  | 409 | 116 | 0.3146 | 0.0006* |
|  | 411 | 103 | 0.3325 | 0.0006* |
|  | 412 | 103 | 0.3587 | 0.0002* |
| Subadult | Overall | 286 | 0.3142 | <0.001* |
|  | 215 | 89 | 0.1357 | 0.2049 |
|  | 243 | 24 | 0.5644 | 0.0041* |
|  | 248 | 106 | 0.3779 | <0.0001* |
|  | 263 | 13 | 0.06965 | 0.0082* |
|  | 386 | 54 | 0.3834 | 0.0042* |
| Adult | Overall | 1110 | 0.3296 | <0.0001* |
|  | 128 | 37 | 0.1613 | 0.3403 |
|  | 136 | 96 | 0.0348 | 0.7364 |
|  | 137 | 112 | 0.0689 | 0.4704 |
|  | 146 | 110 | 0.4141 | <0.0001* |
|  | 148 | 125 | 0.1489 | 0.0974 |
|  | 215 | 100 | 0.1584 | 0.1156 |
|  | 225 | 106 | 0.6646 | <0.0001* |
|  | 234 | 117 | 0.2459 | 0.0075* |
|  | 243 | 48 | 0.5501 | <0.0001* |
|  | 248 | 126 | -0.0473 | 0.5988 |
|  | 263 | 43 | 0.1107 | 0.4796 |
|  | Generic#2 | 90 | 0.2133 | 0.0435* |
| Aged | Overall | 368 | 0.3959 | <0.001* |
|  | 64 | 39 | 0.4201 | 0.0078* |
|  | 75 | 119 | 0.4123 | <0.0001* |
|  | 173 | 77 | -0.1529 | 0.1843 |
|  | 221 | 102 | 0.3835 | <0.0001* |
|  | Generic#1 | 31 | -0.4212 | 0.0183 |
| *Indicates values that are significant (*P* < 0.05). | | | | |
