# Supplementary figures and images for: Linkage between fecal androgen and glucocorticoid metabolites, spermaturia, body weight and onset of puberty in male African lions (Panthera leo)
Source: PLoS One. 2019 Jul 3;14(7):e0217986. doi: 10.1371/journal.pone.0217986 (PMC6609010; doi:10.1371/journal.pone.0217986)

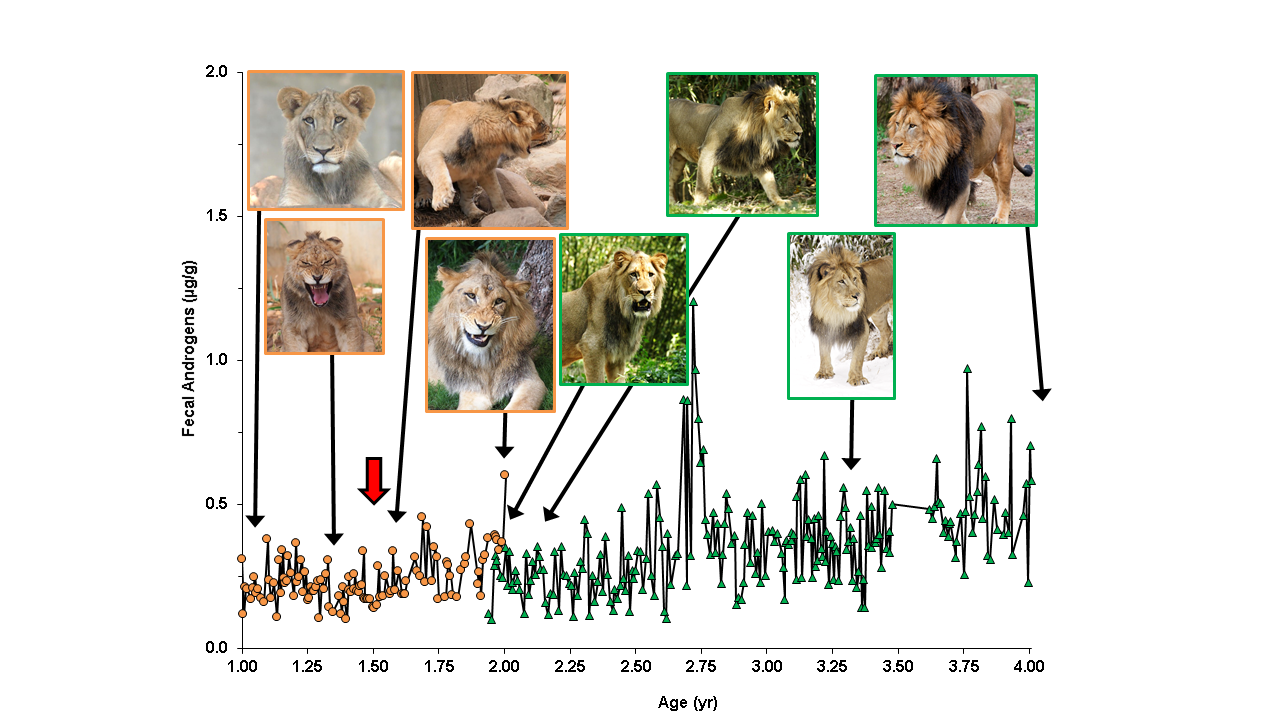

Supplement: S1 Fig — SB409, orange circles; SB248, green triangles. Black arrows point to the age/hormone concentration when the photo was taken. Red arrow indicates when spermatozoa was found in SB409’s urine. SB409 photos courtesy of Karen Shilling, SB248 photos courtesy of Budhan Pukazhenthi and Smithsonian National Zoological Park. (TIF) [file pone.0217986.s002.tif]

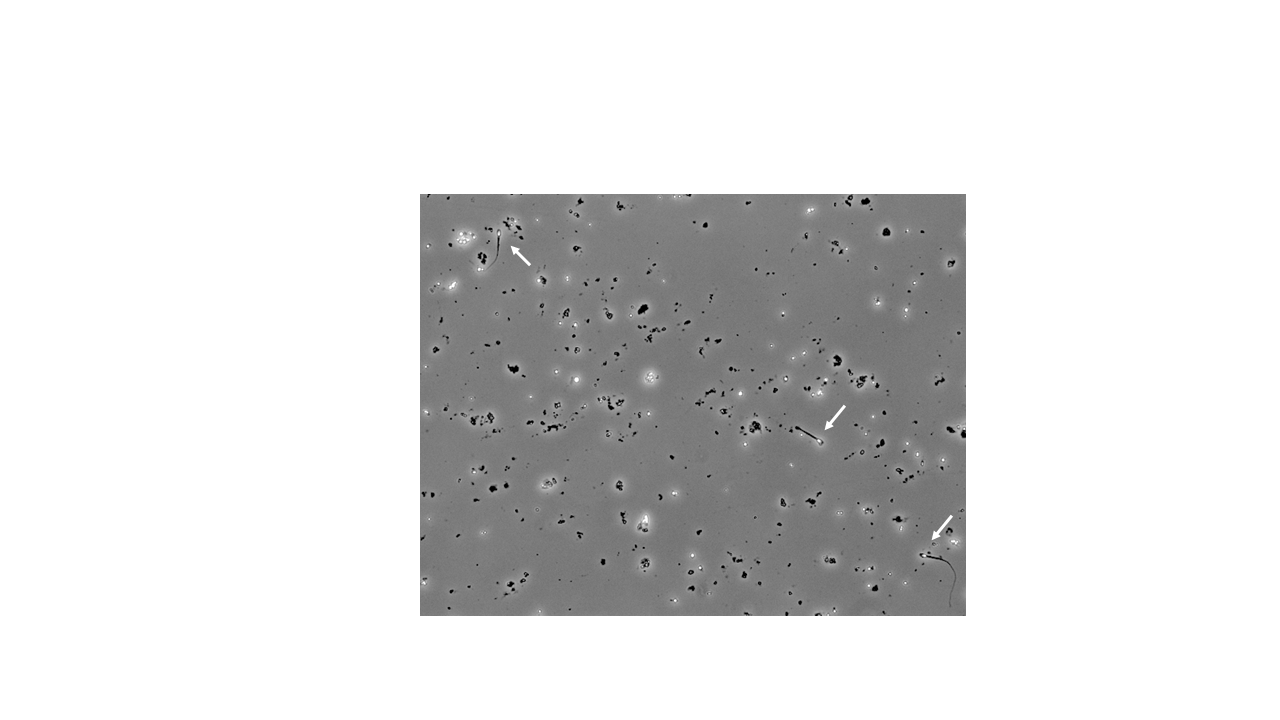

Supplement: S2 Fig — 100x magnification. (TIF) [file pone.0217986.s003.tif]
